# Multi-color single-molecule tracking and subtrajectory analysis for quantification of spatiotemporal dynamics and kinetics upon T cell activation

**DOI:** 10.1038/s41598-017-06960-z

**Published:** 2017-08-01

**Authors:** Yuma Ito, Kumiko Sakata-Sogawa, Makio Tokunaga

**Affiliations:** 1 School of Life Science and Technology, Tokyo Institute of Technology, Nagatsuta-cho, Midori, Yokohama, 226-8501 Japan; 2 Center for Integrative Medical Sciences, RIKEN, Suehiro, Tsurumi, Yokohama, 230-0045 Japan

## Abstract

The dynamic properties of molecules in living cells are attracting increasing interest. We propose a new method, moving subtrajectory analysis using single-molecule tracking, and demonstrate its utility in the spatiotemporal quantification of not only dynamics but also the kinetics of interactions using single-color images. Combining this technique with three-color simultaneous single-molecule imaging, we quantified the dynamics and kinetics of molecules in spatial relation to T cell receptor (TCR) microclusters, which trigger TCR signaling. CD3ε, a component of the TCR/CD3 complex, and CD45, a phosphatase positively and negatively regulating signaling, were each found in two mobility states: faster (associated) and slower (dissociated) states. Dynamics analysis suggests that the microclusters are loosely composed of heterogeneous nanoregions, possibly surrounded by a weak barrier. Kinetics analysis quantified the association and dissociation rates of interactions with the microclusters. The associations of both CD3ε and CD45 were single-step processes. In contrast, their dissociations were each composed of two components, indicating transient and stable associated states. Inside the microclusters, the association was accelerated, and the stable association was increased. Only CD45 showed acceleration of association at the microcluster boundary, suggesting specific affinity on the boundary. Thus, this method is an innovative and versatile tool for spatiotemporal quantification.

## Introduction

Recently, owing to technical developments and an increased number of commercially available instruments, remarkable progress has been made in the elucidation of biological macromolecule dynamics at the single-molecule level^1, 2^, providing fundamental insight into the understanding of molecular functions in living cells^3, 4^. Biological molecules function through interactions with many other proteins such as co-worker and regulatory proteins, resulting in complicated molecular dynamics. Numerous studies have revealed that the behavior of proteins in living cells is heterogeneous^5, 6^. Therefore, it is important to analyze these proteins simultaneously; however, it is difficult to simultaneously capture the movements of different proteins.

T-lymphocyte cell activation in the immune system is a complicated process, in which kinases, phosphatases, and adaptor proteins act simultaneously and/or sequentially. T cell receptor (TCR), composed of TCR subunits and CD3 subunits, recognizes antigenic peptides presented by major histocompatibility complex (MHC) molecules. MHC-TCR complexes induce phosphorylation of the TCR/CD3 complex via a tyrosine kinase, Lck. This causes clustering of signaling molecules and triggers subsequent signal transduction. Lck is activated by a phosphatase, CD45, which dephosphorylates an inhibitory tyrosine of Lck to relieve autoinhibition. Conversely, CD45 negatively regulates signaling by dephosphorylating TCR^7, 8^. Lck shows different activity depending on whether it is diffuse, clustered, or co-clustered with TCR^8^. Thus, CD45 regulates signaling both positively and negatively^7, 8^.

TCR signaling proteins assemble into spatially segregated supramolecular activation clusters (SMAC) at the area of cell contact^9, 10^. A previous study using live cell imaging found that activation causes formation of microclusters of TCR molecules and that the initial stages of the signaling cascade are spatiotemporally controlled on the TCR microclusters^11^. Previous studies using single-molecule tracking on T cell surfaces revealed differences in diffusion coefficients between the inside and outside of lipid rafts^12–14^. However, differences in mobility related to the microclusters were unknown. Furthermore, the kinetics related to the microclusters have not been sufficiently explored.

Here, we have introduced a new method of moving subtrajectory analysis to quantify both dynamics and kinetics spatiotemporally. Use of glass-supported lipid bilayers^15^ via a facile preparation method^16^ enabled us to hold cells onto the surfaces, preserving the mobility of membrane proteins. We applied three-color single-molecule imaging to analyze different kinds of proteins simultaneously. Obtained images were analyzed using moving subtrajectory analysis, and we demonstrated that the new method quantifies not only dynamics but also kinetics in spatial relation to the microclusters.

## Results

### Three-color simultaneous imaging of living cells

**Figure 1:**
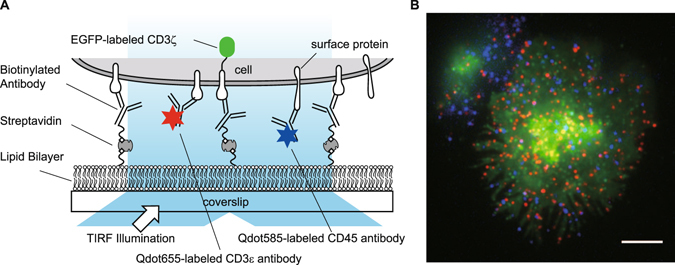
Simultaneous triple-color single-molecule observation using planar lipid bilayers. (**A**) Schematic illustration. (**B**) Representative image of simultaneous three-color single-molecule observation of CD3ζ-EGFP (green), Qdot 655-labeled CD3ε (red), and Qdot 585-labeled CD45 (blue) in living Jurkat cells at 37 °C. Bar, 5 μm.

We visualized the single-molecule dynamics of CD3ε, a subunit of TCR, and CD45. Jurkat cells, an immortalized line of human T cells, stably expressing CD3ζ-EGFP were immobilized onto glass surfaces using biotinylated anti-CD3ε antibodies and planar lipid bilayers on coverslips to preserve the intrinsic mobility of membrane proteins^16^ (Fig. 1A). Therefore, TCR signaling was activated immediately after binding with anti-CD3ε antibodies. CD3ζ-EGFP was used as a marker protein for TCR. CD3ε and CD45 on cell surfaces were fluorescently labeled using antibodies against extracellular domains of CD3ε and CD45 conjugated with quantum dots 655 (Qdot 655) and 585 (Qdot 585), respectively. Fluorescence labeling with Qdots enabled clear visualization of single molecules, as well as tracking for extended periods of time.

Three-color simultaneous imaging of the TCR microcluster, CD3ε, and CD45 was achieved by total internal reflection fluorescence (TIRF) microscopy^17^ using a 488-nm laser beam to excite CD3ζ-EGFP, anti-CD3ε antibody-Qdot 655, and anti-CD45 antibody-Qdot 585 simultaneously (Fig. 1A). The concentration of Qdot-labeled antibodies in staining was optimized for single molecule imaging depending on protein expression level and antibody affinity, and was 3 nM for both anti-CD3ε antibody-Qdot 655 and anti-CD45 antibody-Qdot 585 (Fig. 1B).

**Figure 2:**
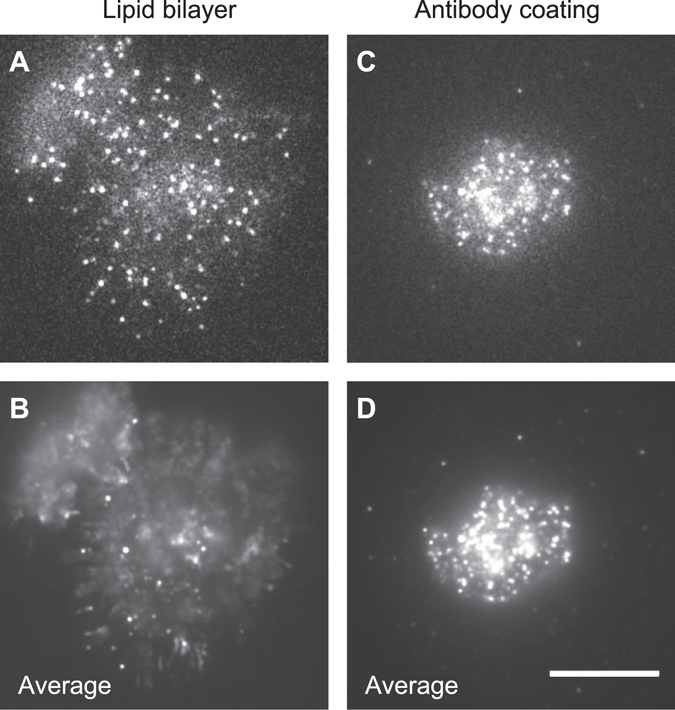
Usage of planar lipid bilayers for examination of molecular mobility in living cells. Single-molecule images of Qdot 585-labeled CD45 in living Jurkat cells on a lipid bilayer (**A,B**) and on an antibody-coated glass surface (**C,D**). Single-frame images (**A,C**) and 200-frame averaged images (**B,D**) recorded at 33 ms/frame are shown. The difference between single-frame images and multi-frame averaged images indicates high molecular mobility. Bar, 10 μm.

Real-time imaging of the surfaces of activated Jurkat cells showed that both CD3ε and CD45 molecules were extensively mobile when the cell was immobilized via planar lipid bilayers (Movie S1)^16^. We examined the fraction of mobile molecules using image averaging (Fig. 2). After averaging over 200 frames (6.67 s), immobile molecules remained as bright spots, whereas mobile molecules blended into the background and disappeared. When Jurkat cells were immobilized via planar lipid bilayers, 70% of the CD45 molecules disappeared (were mobile) (Fig. 2A and B). In contrast, when Jurkat cells were immobilized via anti-CD3ε antibody-coated glass surfaces, only 10% of the CD45 molecules were mobile (Fig. 2C and D). Furthermore, the lipid bilayer prevented nonspecific binding to glass surfaces, as only a few fluorescent spots were detected outside cells. This result clearly indicates that the mobility of molecules on the cell surface is not impaired by immobilization via planar lipid bilayers.

### Single-molecule tracking

**Figure 3:**
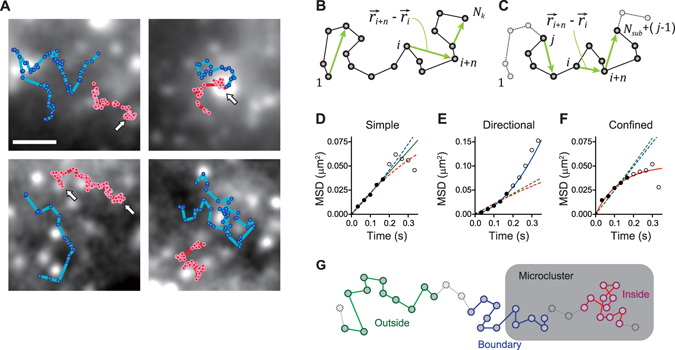
Schematic representation of moving subtrajectory analysis and kinetics analysis. (**A**) Shown is a gallery of single-molecule trajectories of Qdot 585-labeled CD45 (cyan) and Qdot 655-labeled CD3ε (red) superimposed upon 200-frame averaged CD3ζ-EGFP images, which were obtained by analysis of simultaneous triple-color single-molecule movies recorded at 33.33 ms/frame. Arrows indicate trajectories inside TCR microclusters. Bar, 1 μm. (**B**) Standard MSD analysis using single-molecule tracking. (**C**) Moving subtrajectory MSD analysis. All subtrajectories were composed of successive *N*_sub_ spots (*N*_sub_ = 11 in the present study), i.e., (*N*_sub_ − 1) steps were extracted from each trajectory, and their MSD curves were calculated. (**D–F**) Representative examples of sorting of the subtrajectories into the three diffusion types: simple (**D**), directional (**E**), and confined (**F**). After each subtrajectory was fitted to all three equations for the simple (Equation 5, green), directional (Equation 6, blue), and confined (Equation 7, red) diffusion, it was assigned to the diffusion type giving the least residual standard error of the fitting (solid line: assigned, dashed line: not assigned). Within 10 data points of the MSD of a single subtrajectory, the first 5 points (filled circle) were used for the fitting and the later points (open circle) were not. (**G**) Schematic representation of sorting of the subtrajectories of a trajectory into the three location groups: the inside (red), boundary (blue), and outside (green) of the microcluster.

Molecular dynamics were investigated in spatial relation to the microclusters by single-molecule tracking analysis. After intensity-based centroid calculation, trajectories were obtained as coordinates over a series of time steps (Fig. 3A and B). The trajectories of CD3ε and CD45 were superimposed on an average image of the TCR microclusters fluorescently labeled with CD3ζ-EGFP (Fig. 3A). CD3ε and CD45 sometimes followed trajectories along the boundaries of the microclusters. Occasionally, CD3ε entered the microclusters through the boundary, whereas CD45 rarely entered. This finding suggests that the boundary may function as a weak barrier, especially for CD45.

To distinguish the dynamics between the inside and outside of the microclusters, the average images of the microclusters (CD3ζ-EGFP) were binarized and superimposed onto the trajectories (Fig. S1A and B). The movement of the microclusters was negligible in the analysis, as it was much slower than that of the molecules; the movement velocity of the microclusters was 24.9 ± 12.6 nm/s^11^, i.e., 0.8 ± 0.4 nm/frame, whereas most displacements of molecules during a frame were much larger than 10 nm (Fig. S1C–F).

### Standard analysis using single-molecule tracking

The overall dynamics were quantified by calculating the mean square displacement (MSD) (Equation 1), yielding the diffusion coefficient (Equation 2). The overall diffusion coefficient *D* of CD3ε and CD45 was calculated from the slope of the ensemble-averaged MSD curves (Fig. S2, Table S1). High diversity was observed in individual MSD vs. time curves. The individual curves suggest that the molecules have three diffusion types (simple, directional, and confined)^18, 19^ and that the diffusion type of an individual molecule changes over time.

Heterogeneity in mobility was shown using probability distribution functions (PDF, Equation S1, Fig. S1C–F). Bimodal PDF with faster and slower mobility states were obtained for both the inside and outside of the microclusters (Table S1). However, the goodness-of-fit in the PDF analysis is not necessarily sufficient, as the PDF curves are noisy. Moreover, because these standard methods use all spots for each trajectory, they could not analyze temporal and spatial variation of movements along individual trajectories.

### Moving subtrajectory analysis using single-molecule tracking

Aiming to eliminate this problem and to enable quantification of kinetics, we propose a new method, moving subtrajectory (MST) analysis (Fig. 3C). The MSD as a function of space and time (Equation 4) was calculated for a subtrajectory, which is part of a trajectory and is composed of *N*_sub_ successive spots, i.e., (*N*_sub_ − 1) successive steps. In the present study, we set *N*_sub_ = 11. The MSD vs. time curve of each subtrajectory was fitted with three equations, Equations 5–7, describing the two-dimensional simple, directional, and confined diffusion^18, 19^, respectively. Every subtrajectory was sorted into one of the three diffusion types based on the goodness-of-fit (Fig. 3D–F). Subtrajectories were also sorted into one of three groups by location, i.e., the inside, boundary, and outside of the TCR microcluster (Fig. 3G). Subtrajectories that crossed the boundary line were assigned to the boundary group. These analyses were performed for all subtrajectories of all trajectories.

**Figure 4:**
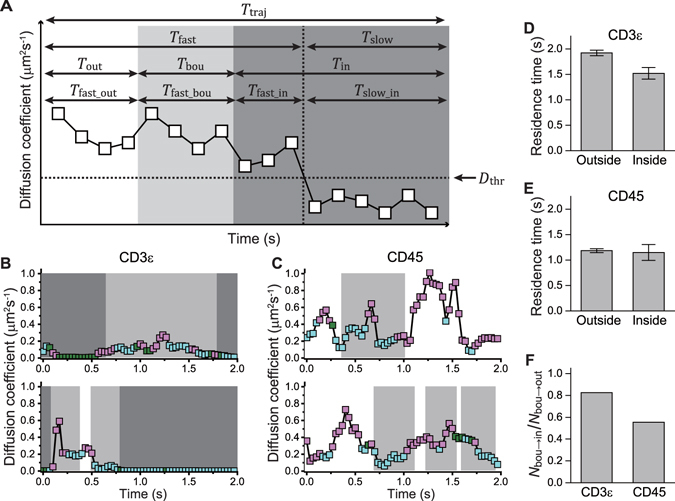
Single-molecule trajectories of CD3ε and CD45 relative to TCR microclusters on the cell membrane of a living Jurkat cell. (**A**) Schematic representation of trajectory durations, residence times, dissociated-state durations, and associated-state durations for lifetime and kinetics analyses. Each square indicates the diffusion coefficient of a single subtrajectory. The locations of the subtrajectories relative to the microclusters are indicated by background colors: inside (white), boundary (light gray), and outside (gray). (**B,C**) Representative time courses of the diffusion coefficient *D* of CD3ε (**B**) and CD45 (**C**) obtained by the moving subtrajectory MSD analysis. The three diffusion types are shown as follows: simple (green square), directional (cyan square), and confined (magenta square). The squares and the background colors are the same as in (**A**). (**D,E**) Lifetimes of the residence times of CD3ε (**D**) and CD45 (**E**) on the inside and outside of the microclusters. (**F**) Ratio for *N*_res_bou–>in_/*N*_res_bou–>out_ between the number of data elements of the residence times at the boundary exiting to the inside and those exiting to the outside.

Moving subtrajectory analysis provides dynamics quantification as a function of space and time. It enables the variations along single trajectories to be followed. For example, variation in both the diffusion coefficient and the diffusion type of single trajectories was visualized in relation to the inside, boundary, and outside of the microclusters (Fig. 4A–C). Furthermore, it yields an abundance of data, whose number is the same as that of the subtrajectories.

### Residence time

Cumulative distributions of trajectory durations of both CD3ε and CD45 were fitted with a double-exponential decay function (Equation 10, Table S2), as they could not be adequately fitted with a single-exponential decay function. The possible causes of the end of the trajectory were: 1) fluorescence quenching by the blinking of Qdots; 2) out-of-focus imaging by three-dimensional diffusion; and 3) failure of single-molecule tracking. Shorter lifetimes *τ*_traj_short_ might be attributed chiefly to blinking, and longer lifetimes *τ*_traj_long_ to out-of-focus imaging and tracking failure.

Residence times on the inside, at the boundary, and on the outside of the microclusters were calculated from the number of subtrajectories that belong continuously to the same regions (Fig. 4A). The number of subtrajectories was used rather than that of steps to ensure consistency with the dynamics and kinetics analyses. Cumulative distributions of all residence times classified by locations for both CD3ε and CD45 were well fitted with a single-exponential decay function (Equation 11, Table S2). Because the residence time is terminated by either exit to another region or by the end of the trajectory, the distributions obey the residence time distribution multiplied by the trajectory duration distribution (Equation 11).

The lifetimes of the residence times have significance not in the absolute values but in the relative values, as they are dependent on the areas and configurations of the regions. The ratios of the lifetime on the outside to that on the inside (*τ*_res_out_/*τ*_res_in_) were 1.27 ± 0.10 and 1.03 ± 0.14 (Table S2) for CD3ε (Fig. 4D) and CD45 (Fig. 4E), respectively. That is, CD3ε remained longer on the outside than CD45. This suggests that movement of CD3ε on the outside involves intermolecular interactions. In contrast, CD45 on the outside may move with relatively less or weaker interactions.

The residence times at the boundary were separated into two subgroups based on whether the subtrajectories exited to the inside or to the outside. For the same reason, relative values are significant, and their lifetime ratios (*τ*_res_bou–>in_/*τ*_res_bou–>out_) were 0.87 ± 0.07 and 0.60 ± 0.06 for CD3ε and CD45, respectively (Table S2). It is noteworthy that the corresponding ratios of the number of data elements (*N*_res_bou–>in_/ *N*_res_bou–>out_) were 0.83 and 0.56 for CD3ε and CD45, respectively (Fig. 4F, Table S2), indicating that the number of subtrajectories of CD45 exiting to the inside is smaller than that exiting to the outside as compared to CD3ε. This corresponds with the observation that the boundary might function as a weak barrier, especially for CD45 (Fig. 3A).

### Quantification of heterogeneous diffusion

**Figure 5:**
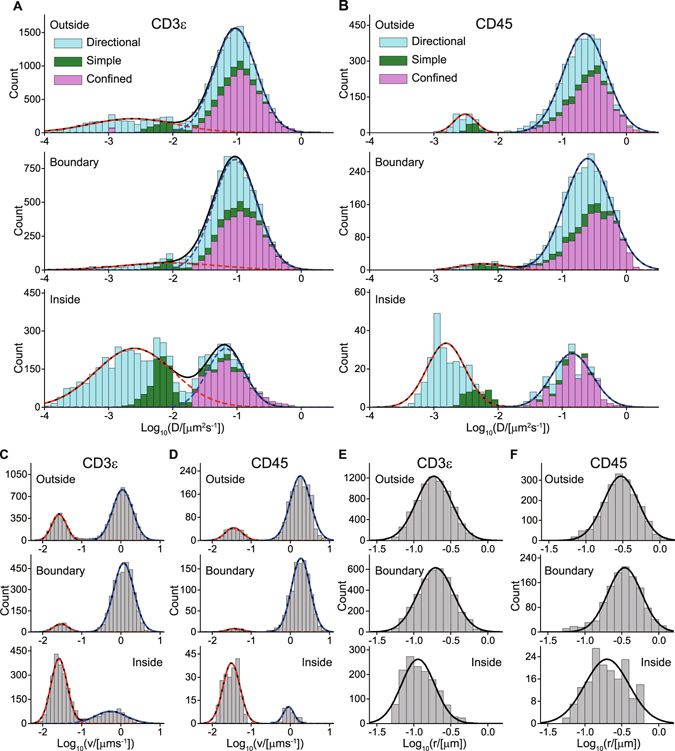
Two mobility states and their properties for CD3ε and CD45 revealed by moving subtrajectory analysis. (**A,B**) Distributions of log_10_(*D/*[μm^2^/s]) on the inside, at the boundary, and on the outside of the microclusters obtained by moving subtrajectory analysis. The three diffusion types are shown as follows: simple (green), directional (cyan), and confined (magenta). The histograms of overall distributions, which were the total of the three diffusion types, were fitted by the dual normal distribution corresponding to the two-state diffusion model (Equation 9, black line) composed of the slower (red dashed line) and faster (blue dashed line) mobility states. The distributions separated into the three diffusion types were also well fitted by the dual normal distribution (Table S4)), (**C**,**D**) Distributions of log_10_(*v*_direc_ */*[μm/s]), where *v*_direc_ is the velocity of the directional movement of the directional diffusion mode (Equation 6). The histograms were fitted by the dual normal distribution (black line), composed of the slower (red dashed line) and faster (blue dashed line) components. (**E,F**) Distributions of log_10_(*r*_conf_ */*[μm]), where *r*_conf_ is the confinement radius of the confined diffusion mode (Equations 7 and 8). The histograms were fitted by the normal distribution (black line).

Moving subtrajectory analysis provided distributions of the diffusion coefficients *D*, distinguishing the locations and the diffusion types (Fig. 5A and B, Table S3). The distributions of log_10_(*D*) of both CD3ε and CD45 were bimodal with the slower and faster mobility states. This separation into two mobility states was performed by fitting Equation 9 to both overall distributions, which were the total of the three diffusion types, and to distributions separated into the simple, directional, and confined diffusion types. Notably, moving subtrajectory analysis provides much clearer peak separation in the diffusion coefficient distributions (Fig. 5A and B) compared to that in the PDF of the standard analysis (Fig. S1C–F).

The overall diffusion coefficients *D*_fast_ of the faster mobility state of CD3ε on the outside, at the boundary, and on the inside were 0.095 ± 0.002, 0.094 ± 0.002, and 0.066 +0.007/−0.006 μm^2^/s, respectively. Those of CD45 were 0.23 ± 0.01, 0.25 ± 0.01, and 0.14 ± 0.02 μm^2^/s, respectively (Fig. 5A and B, Table S3). Those of CD45 on the outside and at the boundary are comparable to the diffusion coefficients of the free streptavidin-conjugated Qdot 655 and Qdot 585 directly bound on the biotinylated lipid bilayer, which were 0.28 +0.07/−0.05 and 0.27 ± 0.02 μm^2^/s, respectively. This indicates that molecules in the faster mobility state are nearly freely mobile, i.e., dissociated.

The overall proportions of the slower mobility states on the outside, at the boundary, and on the inside were roughly 10%, 5%, and 50%, respectively, for both CD3ε and CD45 (Table S3). The overall diffusion coefficients *D*_slow_ of the slower mobility state of CD3ε on the outside, at the boundary, and on the inside were 0.0024 +0.0006/−0.0004, 0.009 +0.043/−0.008, and 0.0025 +0.0004/−0.0003 μm^2^/s, respectively. Those of CD45 were 0.0030 +0.0004/−0.0003, 0.006 +0.004/−0.002, and 0.0015 ± 0.0002 μm^2^/s, respectively. The diffusion coefficients *D*_slow_ were approximately two orders of magnitude lower than *D*_fast_.

The slower mobility state on the inside and outside for both CD3ε and CD45 was mainly composed of the directional diffusion type (Fig. 5A and B), whose velocity *v*_direc_ corresponds to the first peak of the bimodal *v*_direc_ distributions (Fig. 5C and D). This velocity *v*_direc_ at all the locations for both CD3ε and CD45 was approximately 30 ± 10 nm/s (Table S4). This agrees well with the velocity (24.9 ± 12.6 nm/s) of the CD3ζ microcluster movement from the periphery toward the center of primary cultured T cells at steady state^11^. Together with the very low value of *D*_slow_ and the finding of a high proportion of the slower mobility state on the inside, this clearly indicates that molecules in the slower mobility state are associated with the microclusters.

The faster mobility state on the outside and at the boundary for both CD3ε and CD45 was a mix of the three diffusion types, i.e., directional, simple, and confined (Fig. 5A and B), which may be a feature of movement in the narrow space between the microclusters. Supporting this, the confinement radii *r*_conf_ of 0.19 ± 0.01 μm (CD3ε) and 0.30 ± 0.01 μm (CD45) on the outside (Fig. 5E and F, Table S4) were comparable to the dimension of the narrow space remaining (Fig. 3A).

In contrast, the faster mobility state on the inside for both CD3ε and CD45 was mainly composed of the confined diffusion type (Fig. 5A and B), coinciding with the finding that both CD3ε and CD45 remained inside the microclusters for tens of frames once they entered and that the boundary might work as a weak barrier (Fig. 3A). The confinement radii *r*_conf_ of 0.11 ± 0.01 μm (CD3ε) and 0.20 ± 0.02 μm (CD45) on the inside (Fig. 5E and F, Table S4) were comparable to the radius of the microclusters (Fig. 3A). The finding that the faster diffusion coefficients on the inside were smaller than those on the outside and at the boundary (Fig. 5A and B, Table S3) indicates that both CD3ε and CD45 in the faster mobility state inside the microcluster are involved in weak intermolecular interactions.

### Kinetics of mobility state transitions

Moving subtrajectory analysis also enabled kinetics quantification through analysis of the transition between the faster and slower mobility states. We sorted the subtrajectories into slower mobility state, i.e., associated state, and faster mobility state, i.e., dissociated state, according to whether their diffusion coefficients were smaller than a threshold diffusion coefficient *D*_thr_. We used 10^−1.8^ μm^2^/s as *D*_thr_ for both CD3ε and CD45ε, which corresponds to the valley between the two peaks in the distribution. The durations *T*_slow_ and *T*_fast_ were calculated as continuous durations in the same associated state and dissociated state, respectively (Fig. 4A). Cumulative histograms of *T*_slow_ and *T*_fast_ were fitted using a single- or double-exponential decay function. For the same reason given in the residence time analysis (Equation 11), the functions used in the fitting were multiplied by both the trajectory-duration and residence-time distribution functions (Equations 12–15).

**Figure 6:**
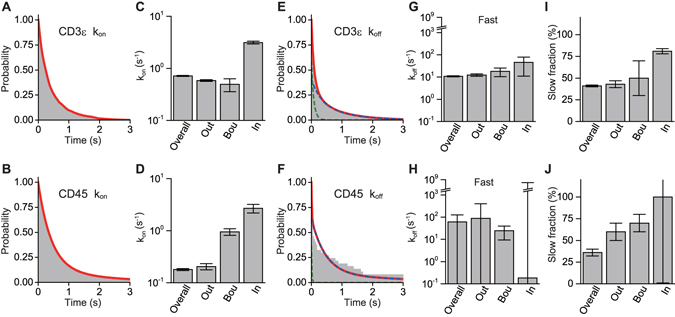
Heterogeneity in the interaction of CD3ε and CD45 with the microclusters is found in dissociation but not in association, as revealed by kinetics analysis. (**A,B**) Cumulative distributions of the overall durations *T*_fast_ of the dissociated state (faster mobility state) of CD3ε (**A**) and CD45 (**B**). Overall data were obtained and analyzed without location classification. The distributions were well fitted by a single-exponential function in terms of the transition (Equation 12, red line), which gave overall association rates *k*_on_. (**C,D**) Association rates of CD3ε (**C**) and CD45 (**D**). Location-classified association rates *k*_on_in_, *k*_on_bou_, and *k*_on_out_ on the inside, at the boundary, and on the outside of the microclusters, respectively, were obtained in the same manner using Equation 13. (**E,F**) Cumulative distributions of the overall durations *T*_slow_ of the associated state (slower mobility state) of CD3ε (**E**) and CD45 (**F**). In contrast to the association rate analysis, the distributions were fitted by a double-exponential function in terms of the transition (Equation 14, red line), which gave slower overall dissociation rates *k*_off_slow_ (blue dashed line) and faster overall dissociation rates *k*_off_fast_ (green dashed line). (**G,H**) Faster dissociation rates of CD3ε (**G**) and CD45 (**H**). Location-classified faster dissociation rates *k*_off_in_fast_, *k*_off_bou_fast_, and *k*_off_out_fast_ on the inside, at the boundary, and on the outside of the microclusters, respectively, were obtained in the same manner using Equation 15. (**I,J**) Fraction *ν* of the slower dissociation of CD3ε (**I**) and CD45 (**J**) (Equations 14 and 15). The relative occurrences of the slower and faster dissociations are *ν* and 1 − *ν*, respectively.

Association rates *k*_on_ were obtained by fitting distributions of the dissociated-state duration *T*_fast_ using a single-exponential decay function (Equations 12 or 13, Fig. 6A and B), meaning that the association reaction is a single-step stochastic process for both CD3ε and CD45. Inside the microclusters, association rates were approximately 5–10 times higher than those on the outside for both CD3ε and CD45 (Fig. 6C and D, Table S5), potentially reflecting the intermolecular interactions on the inside.

At the boundary, the association rate *k*_on_ of CD45 was also higher than that on the outside and relatively similar to that on the inside (Fig. 6D, Table S5). This suggests that CD45, but not CD3ε, interacts with the microclusters at the boundary.

Dissociation rates *k*_off_ were obtained by fitting distributions of the associated-state duration *T*_slow_ using a double-exponential decay function (Equations 14 or 15, Fig. 6E and F) rather than a single-exponential function, and some residual remained in the fitting. This means that the dissociation reaction is a process composed of at least two components for both CD3ε and CD45. The faster dissociation rates *k*_off_fast_ were much higher than the association rate *k*_on_ at all regions for both CD3ε and CD45 (CD3ε: ten to several tens times higher, CD45: one hundred to several hundred times higher) (Fig. 6G and H, Table S5). This indicates that the faster rate represents dissociation from a transient state.

The slower dissociation rates *k*_off_slow_ were not significantly achieved by the fitting due to the large relative values of the standard deviations. This means that all *k*_off_slow_ are much slower than the inverse of the trajectory lifetimes 1/*τ*_traj_long_ (Equations 14 and 15). That is, the dissociation lifetimes 1/ *k*_off_slow_ are much longer than the trajectory lifetimes *τ*_traj_long_ (CD3ε: 1/ *k*_off_slow_ ≫ 0.91 s, CD45: 1/ *k*_off_slow_ ≫ 32 s) (Table S5). This means that the slower rate represents dissociation from a stable associated state. Relative occurrences of the slower and faster dissociations were obtained (Fig. 6I and J, Table S5). The fractions *ν* of slower dissociations were approximately half (CD3ε: 43 ± 4%, CD45: 60 ± 10%) on the outside but represented a large majority on the inside (CD3ε: 81 ± 3%, CD45: 100 ± 100%; the large error for CD45 was caused by the small number of subtrajectories). It is noteworthy that the fraction of slower dissociations largely increased only on the inside for both CD3ε and CD45.

## Discussion

Here, we describe a novel method, moving subtrajectory analysis using single-molecule tracking, which increases accuracy in analysis and enables quantification of kinetics. Using single-color single-molecule images, it quantifies dynamics and kinetics, such as diffusion coefficient, diffusion type and its parameters, association rate, and dissociation rate, as a function of space and time. It enables changes along individual trajectories to be followed.

The moving subtrajectory method markedly reduced analysis errors compared to those in the PDF analysis because it uses several to tens of steps for composing subtrajectories for calculation, whereas the PDF analysis uses single steps. In addition, it uses a large number of data elements equal to the number of subtrajectories. Indeed, histograms of the dynamics parameters showed clear single-peak or bimodal distributions. The accuracy of this analysis has been demonstrated based on the directional-diffusion velocity and the confinement radius.

Kinetics are quantified with spatial relationships by analyzing transitions between different mobility states. This was achieved because the analysis enables mobility changes to be followed along individual trajectories. The association and dissociation rates, as well as the types of reaction mechanisms, were obtained. The association was a single-step process. In contrast, the dissociation was composed of at least two components: the faster dissociation component was faster than association, whereas the slower dissociation component was stable and lasted much longer than a few seconds. This most likely indicates that the associated state is composed of both transient and stable associated states for both CD3ε and CD45. This might be explained by isomerization mechanisms, such as induced fitting or multimerization of the associated state^20^. The discovery of the transient state demonstrates the utility of this method.

Inside the microclusters, approximately half of the molecules of both CD3ε and CD45 were in the slower mobility state. Their diffusion type was almost directional diffusion, and their velocity coincided well with that of the microcluster movement. This clearly indicates that they were associated with the microclusters. The other half of the molecules were in the faster mobility state and relatively freely mobile with weak interactions, as their diffusion coefficient was slightly smaller than that on the outside. Their diffusion type was almost confined diffusion, and the confinement radius was comparable to the radius of the microclusters. These findings clearly indicate that movements are confined within the microclusters. Kinetics analysis showed that the association was accelerated and the stable associated component was increased on the inside compared to the outside, indicating that the interactions of both CD3ε and CD45 with the microclusters are strengthened on the inside.

The present finding of the two mobility states inside the microclusters indicates structural heterogeneity of microclusters. A previous study using live cell imaging revealed that TCR microclusters occasionally split and merged^11^, suggesting that the interactions between the molecules forming the microclusters are not tight but loose. Studies using super-resolution photoactivated localization microscopy (PALM) revealed that TCR molecules and the key signaling molecule (adaptor) LAT existed in separate membrane domains (protein islands) in resting cells, and that they were concatenated after activation^21^. Another study using PALM reported that most LAT molecules were present in very small nanoclusters containing only two to a few molecules in resting cells, and that activation increased the clustering extent by a modest shift toward larger clusters, leaving the very small nanoclusters^22^. Together, the present findings most likely indicate that the TCR microclusters are composed of heterogeneous nanoregions.

Outside the microcluster and at the boundary, in contrast to the inside, most CD3ε and CD45 molecules were present in the faster mobility state. They showed a distinctive feature in that the diffusion type was a mixture of the three diffusion types: directional, simple, and confined. The presence of multiple diffusion types may be explained by the classification of movements in some thin, narrow, irregularly shaped regions as directional, whereas movements in other narrow, irregularly shaped regions were classified as confined.

The other small fraction on the outside and at the boundary was in the slower mobility state. The directional diffusion velocity was equal to that of the microclusters, indicating involvement in interactions. The kinetics analysis also showed that they are involved in interactions based on the existence of slower dissociation. These findings suggest that TCR/CD3 nanoclusters or sub-microclusters are present on the outside and at the boundary, and that CD3ε and CD45 interact with these nanoclusters. The smaller fraction of CD45 in the slower mobility state compared to that of CD3ε again shows fewer or weaker interactions for CD45 compared to CD3ε on the outside.

We observed that CD45 rarely entered microclusters across the boundary, whereas CD3ε occasionally entered. This was quantitatively shown by the ratio *N*_res_bou–>in_/*N*_res_bou–>out_. This result corresponds well with previous studies. It was previously reported that CD45 is mostly excluded from the central SMAC at cell–cell contacts^23, 24^ and from large TCR microclusters with an average diameter of 0.52 ± 0.06 μm^25^. A recent study using fluorescence microscopy of single microclusters *in vitro* showed that the CD45 density in physiological TCR clusters is low because of the exclusion of CD45^8^. These findings support our observation that the boundary might function as a weak barrier against CD45.

Furthermore, the present quantification yielded findings on specific affinity of CD45 at the boundary, i.e., that the association rate *k*_on_ at the boundary was higher than that on the outside. In particular, the ratio of *k*_on_/*k*_off_fast_ for CD45 at the boundary was much higher than that on the outside. A recent study using PALM revealed that recruitment of a downstream signaling molecule, ZAP-70, and TCR activation were localized inside the TCR microclusters except at the boundary^26^. These findings suggest a unique property, i.e., that the boundary of the microcluster functions as a weak barrier and, conversely, has some affinity for CD45.

The diffusion coefficients obtained in the present study agree with those of previous reports: 1) those of the faster mobility state *D*_fast_ of CD45 on the outside and at the boundary (0.225 ± 0.007 and 0.249 ± 0.008 μm^2^/s, respectively) are the same as those of lipid raft-associated glycosylphosphatidylinositol (GPI) proteins CD48 and CD59 (0.23 ± 0.016 and 0.24 ± 0.048 μm^2^/s, respectively) determined using Alexa647-labeled Fab fragments^13^; and 2) those of the faster mobility state *D*_fast_ of CD3ε and CD45 on the inside (0.066 +0.007/−0.006 and 0.14 ± 0.002 μm^2^/s, respectively) are almost the same as those of CD3ε and CD45 on unstimulated naive CD4^+^CD25^−^ T lymphocytes (0.06 ± 0.01 and 0.087 ± 0.012 μm^2^/s, respectively)^13^. The diffusion coefficients *D*_fast_ of CD45 were 2 to 2.5 times larger than those of CD3ε at all locations. This means that movement of CD45 involves more rapid interactions than movement of CD3ε. This may be explained by the finding that the sum of the overall association and dissociation rate *k*_on_ + *k*_off_fast_ of CD45 was 5 times larger than that of CD3ε, as it provides a measure of reaction velocity.

Furthermore, it has been reported that Qdot labeling does not affect the diffusion of membrane proteins^27^. Indeed, the diffusion coefficients of free streptavidin-conjugated Qdot 655 and Qdot 585 on the lipid bilayer in the present study were 0.28 +0.07/−0.05 and 0.27 ± 0.02 μm^2^/s, respectively. Therefore, the potential influence of the difference between Qdot 655 and Qdot 585, which were used for labeling CD3ε and CD45, respectively, is not a cause for concern. However, in the interest of accuracy, we compared the diffusion coefficients of CD3ε and CD45 not by the absolute values but rather by the relative values, for example, by comparing the ratios between the different states and locations.

We stimulated Jurkat T cells using the CD3 antibody. Costimulation with CD3 and CD28 is required for full activation of T cells, and differences in downstream activation of signaling pathways have been revealed between CD3 stimulation and CD3/CD28 costimulation, such as in the PKC and MAPK/ERK pathways^28, 29^. Differences between Jurkat cells and primary T cells in signaling activation have been also investigated, with observation of differences caused by PTEN deficiency^30^ and differences in PI3K activation mediated via Ras^31^. It has been reported that Jurkat cells showed centripetal movements upon activation, similarly to primary T cells^32^. The present method should enable further elucidation of these mechanisms.

In the present study, we demonstrated that moving subtrajectory analysis quantitatively produces a considerable amount of spatiotemporal information on dynamics and kinetics from single-color single-molecule images. Combined use of multi-color single-molecule imaging enables the analysis of different kinds of proteins simultaneously and yields much more information. Thus, the present method is innovative and opens new avenues for quantification to elucidate molecular mechanisms of function.

## Materials and Methods

### Reagents and cell preparation

DOPC (1,2-dioleoyl-*sn*-glycero-3-phosphocholine) was purchased from Avanti Polar Lipids (AL, USA). *N*-((6-(biotinoyl)amino)hexanoyl)-1,2-dihexadecanoyl-*sn*-glycero-3-phosphoethanolamine (Biotin-X-DHPE) and streptavidin were purchased from Invitrogen (Japan). Monoclonal antibody against CD45 (MEM-28) was obtained from Abcam (Japan). Monoclonal antibody against CD3ε (HIT3a; BD Pharmingen, Japan) was biotinylated using NH_2_-reactive biotin (Dojindo Molecular Tech., Japan). Qdot 655 and Qdot 585 were conjugated to anti-CD3ε and -CD45 antibodies, respectively, using the Qdot Antibody Conjugation Kit (Invitrogen) according to the manufacturer’s instructions. CD3ζ-EGFP cDNA was a gift from Drs. T. Yokosuka and T. Saito (RIKEN, Yokohama, Japan). cDNA was subcloned into the Gateway destination vector pEF5/FRT/V5-DEST (Invitrogen). Flp-In Jurkat T cells (Invitrogen) were cultured in RPMI 1640 medium supplemented with 10% fetal bovine serum, 2 mM l-glutamine, 50 U/ml penicillin, and 50 μg/ml streptomycin at 37 °C in a 5% CO_2_ atmosphere. Subcloned cDNA was transfected by electroporation into Flp-In Jurkat cells to generate a stably expressing cell line as previously described^16^. Cells were fluorescently labeled on the outer surfaces with Qdot-conjugated antibodies for 5 min before imaging.

### Planar lipid bilayers

Supported planar bilayers were formed by liposome fusion on 35-mm glass-bottom dishes (MatTek, MA, USA) as described previously with minor modifications^16^. DOPC lipid films containing 0.1 mol% Biotin-X-DHPE were rehydrated in TBS buffer (50 mM Tris, 137 mM NaCl, 2.7 mM KCl, pH 7.5) with 2% octyl *β*-d-glucopyranoside (Sigma-Aldrich, Japan) and sonicated with a water-bath sonicator (UT-104; Sharp, Japan) for 30 min. The liposome suspension was filtered with a 0.22-μm filter (Millipore, Japan) and dialyzed for 36 h at 4°C. Liposomes were deposited on a glass surface, and then streptavidin and biotinylated anti-CD3ε antibodies were sequentially conjugated with the lipid bilayers. Imaging medium (25 mM HEPES and MEM without phenol red, riboflavin, and folic acid) was added to the lipid bilayers before observation.

### Single-molecule microscopy

For T cell stimulation, the CD3ζ-EGFP expressing Jurkat cells were allowed to attach to the lipid bilayers at 37 °C for 2 min prior to imaging. For stimulation by antibody coating as a control experiment, anti-CD3ε-antibody-coated surfaces were used rather than the lipid bilayers. The surfaces were prepared by adsorbing 1 μg/mL anti-CD3ε antibody solution overnight at 4°C onto a coverslip^30^.

Cells were imaged with a custom-built TIRF and HILO (highly inclined and laminated optical sheet) microscope setup^17, 33^ based on an inverted microscope (IX-81, Olympus, Japan) equipped with an infinity-corrected objective (PlanApo 100× NA 1.45 oil TIRFM, Olympus, Japan). A beam from a solid-state laser (488 nm and 20 mW; Sapphire 488-20-OPS; Coherent, Japan) was used for fluorescence illumination. Optical filters (custom-order, Olympus) included a dichroic mirror (DM488) and emission filters (Em 495-545 for EGFP, Em 569-624 for Qdot 585, and Em 650-705 for Qdot 655). Images were captured with three electron-multiplying charge-coupled device (EMCCD) cameras (C9100-13, Hamamatsu Photonics, Japan) controlled by AQUACOSMOS software (Hamamatsu Photonics). Specimens were observed at 37 °C using a temperature control system with a stage top incubator and an objective heater (IBC-IU2-TOP/-CB/-LH, MI-IBC-IU2, Tokai Hit, Japan).

### Image analysis

After noise reduction was applied to the acquired images, differences in magnification, shift, and rotation among color channels were corrected with a square lattice using ImageConverter (Olympus Software Technologies, Japan). Images for CD3ζ-EGFP were averaged over 200 frames (33.33 ms/frame). Averaged images were processed with a Fourier transform filter to reduce background and then binarized manually^12^. Binary images were superimposed on the single-molecule images of CD3ε and CD45, and these were used as the regions of the TCR microclusters.

### Single-molecule tracking

Trajectories of individual molecules were determined using the Particle Tracker plug-in for ImageJ^34^. Each trajectory was composed of a series of spots, and intervals between the adjacent spots were steps. The time interval of adjacent spots was the frame interval Δ*t*, 33.33 ms.

### Standard analysis methods

#### Mean square displacement analysis

The mean square displacement (MSD) of the *k*-th trajectory (*k* = 1,…, *N*_traj_), where *N*_traj_ is the number of the trajectories, was calculated according to the definition^35, 36^:

1 $${\rho }_{k}(n{\rm{\Delta }}t)=\frac{1}{{N}_{k}-n}\,\sum _{i=1}^{{N}_{k}-n}{({\overrightarrow{r}}_{i+n}-{\overrightarrow{r}}_{i})}^{2},\phantom{\rule{7ex}{0ex}}n=1,\,\ldots ,{N}_{k}-1\,$$

where ${\rho }_{k}(n{\rm{\Delta }}t)$ is the MSD of duratio*n n*Δ*t*, *N*_*k*_ is the number of spots on the *k*-th trajectory, and ${\overrightarrow{r}}_{i}$ = (*x*_i_, *y*_i_) is the position of the *i* ^th^ spot. This definition uses all available displacements of the duratio*n n*Δ*t* (Fig. 3B). The averaged MSD *ρ*¯(*n*Δ*t*) was calculated by averaging *ρ*_*k*_(*n*Δ*t*) for all the trajectories (*k* = 1,…, *N*_traj_). The diffusion coefficient *D* was determined by fitting the averaged MSD curve, $\bar{\rho }(n{\rm{\Delta }}t)$ vs. *n*Δ*t*, with the following equation:

2 $$\rho (t)=4Dt,$$

where *ρ*(*t*) is the theoretical function of MSD for simple diffusion against time *t* = *n*Δ*t*.

#### Analysis using probability distribution function

The probability distribution function PDF(r, *t*) is defined as a probability density as follows: ${\rm{PDF}}(r,t){\rm{d}}r$ is the probability that a displacement during time interval *t* is found between *r* and *r* + d*r* ^37, 38^. In the case of two-dimensional simple diffusion, theoretical PDF(*r*, *t*) is derived as follows:

3 $$\begin{array}{rcl}{\rm{PDF}}(r,t){\rm{d}}r & = & \frac{1}{{(\sqrt{4\pi Dt})}^{2}}\exp (-\frac{{r}^{2}}{4Dt})2\pi r{\rm{d}}r\\ \therefore {\rm{PDF}}(r,t) & = & \frac{r}{2Dt}\exp (-\frac{{r}^{2}}{4Dt})\end{array},$$

meaning that it follows the Rayleigh distribution.

### Moving subtrajectory analysis

#### Moving subtrajectory MSD analysis

MSD analysis was performed using a “subtrajectory” composed of *N*_sub_ spots (*N*_sub_ = 11 in the present study), i.e., (*N*_sub_ − 1) steps (Fig. 3C). The MSD of the *j*-th subtrajectory (*j* = 1,…, *N*_*k*_ − *N*_sub_ + 1) of the *k*-th trajectory (*k* = 1,…, *N*_traj_) was calculated as follows:

4 $${\rho }_{j,k}(n{\rm{\Delta }}t)=\frac{1}{{N}_{{\rm{s}}{\rm{u}}{\rm{b}}}-n}\,\sum _{i=j}^{{N}_{{\rm{s}}{\rm{u}}{\rm{b}}}+(j-1)-n}{({\overrightarrow{r}}_{i+n}-{\overrightarrow{r}}_{i})}^{2}.\phantom{\rule{7ex}{0ex}}n=1,\ldots ,{N}_{{\rm{s}}{\rm{u}}{\rm{b}}}-1$$

Every subtrajectory was sorted into three diffusion types: simple, confined, and directional. MSD curves, ${\rho }_{j,k}(n{\rm{\Delta }}t)$ vs. *n*Δ*t*, were fitted on the *n* = 1 to *N*_fit_ values (${N}_{{\rm{fit}}}\le {N}_{{\rm{sub}}}$, *N*_fit_ = 5 in the present study) with the three following equations describing the two-dimensional simple, directional, and confined diffusion, respectively^18, 19^:

5 $${\rho }_{{\rm{s}}{\rm{i}}{\rm{m}}{\rm{p}}{\rm{l}}{\rm{e}}}(t)=4Dt,\phantom{\rule{10ex}{0ex}}$$

6 $${\rho }_{{\rm{d}}{\rm{i}}{\rm{r}}{\rm{e}}{\rm{c}}}(t)=4Dt+{v}_{{\rm{d}}{\rm{i}}{\rm{r}}{\rm{e}}{\rm{c}}}^{2}{t}^{2},\phantom{\rule{1.5ex}{0ex}}$$

7 $${\rho }_{{\rm{c}}{\rm{o}}{\rm{n}}{\rm{f}}}(t)=4D\tau \,(1-{e}^{-t/\tau }),$$

where *v*_direc_ is the magnitude of the velocity of directional diffusion, *r*_conf_ is the confinement radius, and *τ* is a time constant defined as follows:

8 $$\tau =\frac{{r}_{{\rm{conf}}}^{2}}{3D}.\,$$

It is noteworthy that Equation 7 approaches Equation 5 in the vicinity of the origin ($|t|\ll \tau$):

$${\rho }_{{\rm{c}}{\rm{o}}{\rm{n}}{\rm{f}}}(t)=4D\tau \,(1-(1-\frac{t}{\tau }))=4Dt.\,$$

Each subtrajectory was assigned to a diffusion type based on the residual standard errors of the fitting (Fig. 3D–F).

#### Analysis using histograms of diffusion coefficients

Histograms of the logarithms of diffusion coefficients obtained by the moving subtrajectory MSD analysis were fitted by the dual normal distribution corresponding to the two-state diffusion model,

9 $$\begin{array}{ccc}f(u) & = & {N}_{{\rm{d}}{\rm{a}}{\rm{t}}{\rm{a}}}{\rm{\Delta }}u\,[\xi \frac{1}{\sqrt{2\pi {\sigma }_{{\rm{s}}{\rm{l}}{\rm{o}}{\rm{w}}}^{2}}}\exp (-\frac{{(u-{{\rm{l}}{\rm{o}}{\rm{g}}}_{10}{D}_{{\rm{s}}{\rm{l}}{\rm{o}}{\rm{w}}})}^{2}}{2{\sigma }_{{\rm{s}}{\rm{l}}{\rm{o}}{\rm{w}}}^{2}})\\  &  & +(1-\xi )\frac{1}{\sqrt{2\pi {\sigma }_{{\rm{f}}{\rm{a}}{\rm{s}}{\rm{t}}}^{2}}}\exp (-\frac{{(u-{{\rm{l}}{\rm{o}}{\rm{g}}}_{10}{D}_{{\rm{f}}{\rm{a}}{\rm{s}}{\rm{t}}})}^{2}}{2{\sigma }_{{\rm{f}}{\rm{a}}{\rm{s}}{\rm{t}}}^{2}})],\end{array}$$

where *u* = log_10_(*D*/[*μ*m^2^/s]), *ξ* is a relative occurrence, *N*_data_ is the number of data elements, Δ*u* is the histogram bin-width, and *D*_slow_ and *D*_fast_ are diffusion coefficients of slower and faster mobility states, respectively.

#### Sorting of subtrajectories by location

Using the superimposed binary images of TCR microclusters (CD3ζ-EGFP), every subtrajectory was sorted into three location groups: the inside, boundary, and outside of the microcluster (Fig. 3G). Subtrajectories that crossed the boundary line were assigned to the boundary group.

### Lifetime analysis

#### Lifetime of trajectory durations

Cumulative histograms of trajectory durations *T*_traj_ (Fig. 4A) were fitted by a double-exponential decay function,

10 $$f(t)={N}_{{\rm{d}}{\rm{a}}{\rm{t}}{\rm{a}}}{\rm{\Delta }}t\,[\xi \exp (-\frac{t}{{\tau }_{{\rm{t}}{\rm{r}}{\rm{a}}{\rm{j}}{\rm{\_}}{\rm{s}}{\rm{h}}{\rm{o}}{\rm{r}}{\rm{t}}}})+(1-\xi )\,\exp (-\frac{t}{{\tau }_{{\rm{t}}{\rm{r}}{\rm{a}}{\rm{j}}{\rm{\_}}{\rm{l}}{\rm{o}}{\rm{n}}{\rm{g}}}})],$$

where the variable *t* represents *T*_traj_, *N*_data_ is equal to the number of trajectories *N*_traj_, Δ*t* is the histogram bin-width (i.e., the frame interval), *ξ* is a relative occurrence, and *τ*_traj_short_ and *τ*_traj_long_ are lifetimes.

#### Lifetime of residence times

Residence times *T*_res_in_, *T*_res_bou_, *T*_res_out_ on the inside, boundary, and outside of the microclusters were calculated as Δ*t* multiplied by the number of subtrajectories that belong continuously to the same inside, boundary, and outside group, respectively (Fig. 4A). The residence time *T*_res_bou_ at the boundary was separated into two subgroups, i.e., *T*_res_bou–>in_ and *T*_res_bou–>out_, according to whether the subtrajectories exited to the inside or outside, respectively.

Cumulative histograms of residence times *T*_res_*_ (*represents in, bou, out, bou–> in, or bou–> out) were fitted by a single-exponential decay function of *T*_res_*_,

11 $$f(t)={N}_{{\rm{data}}}{\rm{\Delta }}t\,\exp (-\frac{t}{{\tau }_{{\rm{res}}\_\ast }})\,[\xi \,\exp (-\frac{t}{{\tau }_{{\rm{traj}}\_{\rm{short}}}})+(1-\xi )\exp (-\frac{t}{{\tau }_{{\rm{traj}}\_{\rm{long}}}})],$$

where the variable *t* represents *T*_res_*_, *τ*_traj_*_ is a lifetime, and *N*_data_ is equal to *N*_res_*_. During the fitting procedure, the relative occurrence *ξ* is variable, whereas the lifetimes *τ*_traj_short_ and *τ*_traj_long_ are the constants obtained by the trajectory duration analysis with Equation 10.

#### Kinetics analysis

The subtrajectories were sorted into two categories, i.e., associated state (slower mobility state) and dissociated state (faster mobility state), according to whether their diffusion coefficients were smaller than a threshold diffusion coefficient *D*_thr_. The threshold *D*_thr_ was determined as the boundary between the two peaks of the slower and faster mobility states in the distribution of log_10_(*D/*[*μ*m^2^/s]). Durations *T*_slow_ and *T*_fast_ were calculated as durations where the frame interval Δ*t* was multiplied by *N*_slow_ and *N*_fast_, which are the number of subtrajectories that belong continuously to the same associated state and dissociated state, respectively (Fig. 4A). The durations *T*_slow_ and *T*_fast_ were further separated into three groups, i.e., *T*_slow_in_, *T*_slow_bou_, and *T*_slow_out_, and *T*_fast_in_, *T*_fast_bou_, and *T*_fast_out_, based on the location groups, i.e., inside, boundary, and outside, respectively.

#### Overall association rate

The overall association rates, i.e., association rates without location classifications, of the transitions from the dissociated state to the associated sate were obtained by fitting cumulative histograms of the dissociated-state durations *T*_fast_ using a single-exponential decay function in terms of the transition,

12 $$f(t)={N}_{{\rm{data}}}{\rm{\Delta }}t\,\exp (-{k}_{{\rm{on}}}\,t)\,[\xi \,\exp (-\frac{t}{{\tau }_{{\rm{traj}}\_{\rm{short}}}})+(1-\xi )\exp (-\frac{t}{{\tau }_{{\rm{traj}}\_{\rm{long}}}})],\,$$

where the variable *t* represents *T*_fast_, and *k*_on_ is an overall association rate. During the fitting procedure, the relative occurrence *ξ* was variable, whereas the lifetimes *τ*_traj_short_ and *τ*_traj_long_ were the constants obtained by the trajectory duration analysis (Equation 10).

#### Location-classified association rate

Association rates with location classifications were obtained by fitting cumulative histograms of the dissociated-state durations *T*_fast_*_ (*represents in, bou, or out) using a single-exponential decay function in terms of the transition,

13 $$\begin{array}{rcl}f(t) & = & {N}_{{\rm{data}}}{\rm{\Delta }}t\,\exp (-{k}_{{\rm{on}}\_\ast }\,t)\,\,\exp (-\frac{t}{{\tau }_{{\rm{res}}\_\ast }})\,[\xi \,\,\exp (-\frac{t}{{\tau }_{{\rm{traj}}\_{\rm{short}}}})\\  &  & +(1-\xi )\exp (-\frac{t}{{\tau }_{{\rm{traj}}\_{\rm{long}}}})],\end{array}$$

where the variable *t* represents *T*_dis_*_, and *k*_on_*_ is an association rate. During the fitting procedure, the relative occurrence *ξ* was variable, whereas the lifetimes *τ*_res_*_, *τ*_traj_short_, and *τ*_traj_long_ were the constants obtained by the residence time analysis (Equation 11) and trajectory duration analysis (Equation 10).

#### Overall dissociation rate

Overall dissociation rates, i.e., dissociation rates without location classifications, of the transitions from the associated state to the dissociated state were obtained by fitting cumulative histograms of the associated state durations *T*_slow_ using a double-exponential decay function in terms of the transition,

14 $$\begin{array}{rcl}f(t) & = & {N}_{{\rm{data}}}{\rm{\Delta }}t\,[\nu \,\exp (-{k}_{{\rm{off}}\_{\rm{slow}}}\,t)+(1-\nu )\exp (-{k}_{{\rm{off}}\_{\rm{fast}}}\,t)]\\  &  & \,\times [\xi \,\exp (-\frac{t}{{\tau }_{{\rm{traj}}\_{\rm{short}}}})+(1-\xi )\exp (-\frac{t}{{\tau }_{{\rm{traj}}\_{\rm{long}}}})],\end{array}$$

where the variable *t* represents *T*_slow_, and *k*_off_slow_ and *k*_off_fast_ are the overall dissociation rates. During the fitting procedure, the relative occurrences *ν* and *ξ* were variable, whereas the lifetimes *τ*_traj_short_ and *τ*_traj_long_ were the constants obtained by the trajectory duration analysis (Equation 10).

#### Location-classified dissociation rate

Dissociation rates with location classifications were obtained by fitting cumulative histograms of the associated-state durations *T*_slow_*_ (*represents in, bou, or out) using a double-exponential decay function in terms of the transition,

15 $$\begin{array}{rcl}f(t) & = & {N}_{{\rm{data}}}{\rm{\Delta }}t\,[\nu \,\exp (-{k}_{{\rm{off}}\_\ast \_{\rm{slow}}}\,t)+(1-\nu )\exp (-{k}_{{\rm{off}}\_\ast \_{\rm{fast}}}\,t)]\\  &  & \times \exp (-\frac{t}{{\tau }_{{\rm{res}}\_\ast }})\,[\xi \,\exp (-\frac{t}{{\tau }_{{\rm{traj}}\_{\rm{short}}}})+(1-\xi )\exp (-\frac{t}{{\tau }_{{\rm{traj}}\_{\rm{long}}}})],\end{array}$$

where the variable *t* represents *T*_slow_*_, and *k*_off_*_slow_ and *k*_off_*_fast_ are dissociation rates. During the fitting procedure, the relative occurrences *ν* and *ξ* were variable, whereas the lifetimes *τ*_res_*_, *τ*_traj_short_, and *τ*_traj_long_ were the constants obtained by the residence time analysis (Equation 11) and trajectory duration analysis (Equation 10).

## Electronic supplementary material

Video 1

Supplementary Information
