# Supplementary material for: Multi-color single-molecule tracking and subtrajectory analysis for quantification of spatiotemporal dynamics and kinetics upon T cell activation
Source: Sci Rep. 2017 Aug 1;7:6994. doi: 10.1038/s41598-017-06960-z (PMC5539329; doi:10.1038/s41598-017-06960-z)
Supplement: Supplementary file 2 — Supplementary Information [file 41598_2017_6960_MOESM2_ESM.pdf]

Supplementary Information

Multi-color single-molecule tracking and subtrajectory analysis for quantification of spatiotemporal dynamics and kinetics upon T cell activation

Yuma Ito[1], Kumiko Sakata-Sogawa[1,2,*], Makio Tokunaga[1,2,*]

[1]*School of Life Science and Technology, Tokyo Institute of Technology, Nagatsuta-cho, Midori, Yokohama 226-8501, Japan*
[2]*Center for Integrative Medical Sciences, RIKEN, Suehiro, Tsurumi, Yokohama 230-0045, Japan*
*Correspondence and requests for materials should be addressed to M.T. (email: mtoku@bio.titech.ac.jp), and K.S. (email: ksogawa@hilo.bio.titech.ac.jp)

Supplementary information comprises:

Supplementary Movie Legends
Supplementary Results and Discussion
Supplementary Methods
Supplementary References
Supplementary Figures S1 and S2
Supplementary Tables S1–S5

Supplementary Movie Legends

Movie S1

Simultaneous three-color single-molecule imaging of CD3ζ-EGFP (green), Qdot 655-labeled CD3ε (red), and Qdot 585-labeled CD45 (blue) in living Jurkat cells at 37°C at a frame rate of 30 Hz.

Supplementary Results and Discussion

1. Standard analysis using single-molecule tracking

1.1 Ensemble-averaged mean square displacement

Single-molecule trajectories of CD3ε and CD45 superimposed upon a binary average image of the TCR microclusters (CD3ζ) were used to quantify the dynamics (Fig. S1A and B). The overall diffusion coefficient D was obtained by calculating ensemble-averaged mean square displacements (MSD) as a function of time (Equation 1, Fig. S2). The ensemble-averaged MSD vs. time curves of CD3ε and CD45 were nearly linear during the first 0.3–0.4 s, i.e., approximately ten frames within the margin of error. The diffusion coefficient D calculated from the slope of MSD (Equation 2) was 0.0911 ± 0.0006 μm^2/s for CD3ε (95% confidence interval) and 0.270 ± 0.004 μm^2/s for CD45 (Table S1).

This result that the overall diffusion coefficient D of CD3ε was three times smaller than that of CD45 does not correspond with the result using moving subtrajectory analysis (Fig. 5). In the case of heterogeneous movements, overall quantification should be used with caution.

1.2 Two mobility states found inside the TCR microclusters by PDF analysis

Information on heterogeneous mobility was obtained by analysis using probability distribution functions (PDF; Fig. S1C–F). Curve fitting showed that a single-state diffusion model (Equation 3) was not sufficient to explain the PDFs of both CD3ε and CD45. In contrast, a two-state diffusion model (Equation S1) provided better fits to the PDFs of both CD3ε and CD45, yielding diffusion coefficients D_{fast} and D_{slow} for both the inside and outside the microclusters.

The three-state diffusion model[1] can improve goodness-of-fit. However, since the subtrajectory analysis showed that the diffusion coefficients were distributed in two peaks, the two-state diffusion model is judged to be appropriate for the present study.

The diffusion coefficients D_{fast} of the faster mobility state of CD3ε and CD45 inside the microclusters were 0.057 ± 0.004 μm^2/s (relative occurrence 66%) and 0.178 ± 0.016 μm^2/s (77%), respectively (Table S1). It is noteworthy that the diffusion coefficients D_{fast} of both CD3ε and CD45 inside the microclusters are similar to those outside the microclusters. Therefore the faster mobility state inside the microclusters is similar to the mobility state outside the

microclusters.

The diffusion coefficients D_{slow} of the slower mobility state inside the microclusters of CD3ε and CD45 were 0.0041 ± 0.0006 (34%) and 0.0091 ± 0.0015 μm^2/s (23%), respectively (Table S1). They were 14 and 20 times smaller than D_{fast} of the faster mobility state, respectively. This result suggests that intermolecular interactions largely reduce the diffusion coefficients. In addition, the relative occurrence of the slower mobility against the faster mobility is larger on the inside of the microclusters compared with that on the outside. This suggests that the slower mobility state reflects molecular interactions with the microclusters.

Supplementary Methods

1. Standard analysis methods

1.1 Fitting analysis using probability distribution function

The histogram of displacements $\Delta r = |\vec{r}_{i+1} - \vec{r}_i|$ during the frame interval Δt was obtained from all trajectories in a data set. Then, $\mathrm{PDF}(\Delta r, \Delta t)$ was calculated by dividing the histogram by N_{total}, where N_{total} is the total number of displacements Δr in all trajectories. $\mathrm{PDF}(\Delta r, \Delta t)$ was fitted by Equation 3 for the single-state diffusion model and by the following equation for the two-state diffusion model,

$$\mathrm{PDF}_2(r,t) = \xi \frac{r}{2D_{\mathrm{slow}}t} \exp\left(-\frac{r^2}{4D_{\mathrm{slow}}t}\right) + (1-\xi)\frac{r}{2D_{\mathrm{fast}}t}\exp\left(-\frac{r^2}{4D_{\mathrm{fast}}t}\right), \quad \text{(S1)}$$

where ξ is a relative occurrence and D_{slow} and D_{fast} are the diffusion coefficients of the two states.

Supplementary References

1. Gebhardt, J. C. *et al.* Single-molecule imaging of transcription factor binding to DNA in live mammalian cells. *Nat. Methods* 10, 421-426 (2013).

Supplementary Figures

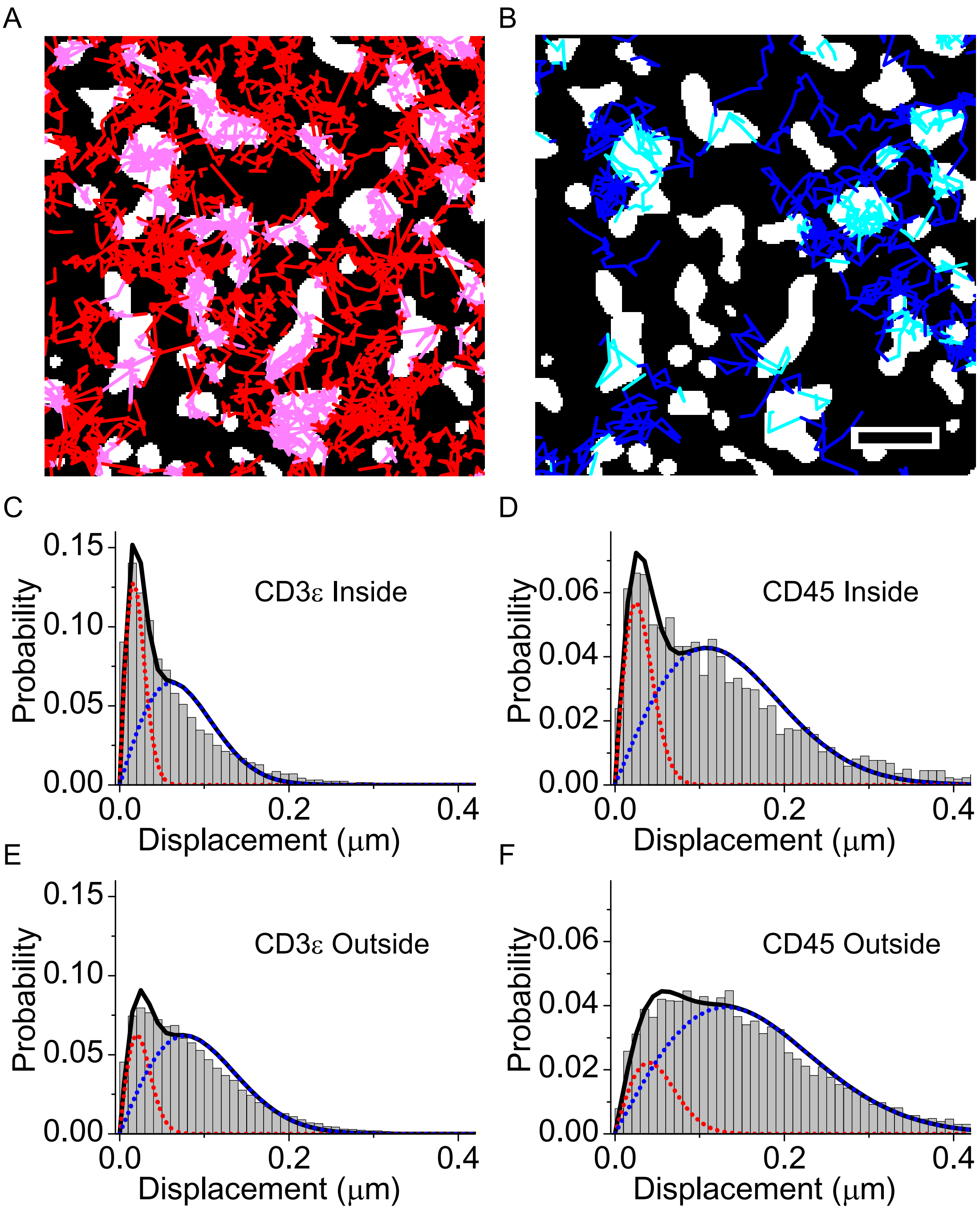

Figure S1. Two different mobility states of CD3ε and CD45 in the microclusters using probability distribution function (PDF) of standard single-molecule tracking analysis. (A, B) Trajectories of CD3ε (A) and CD45 (B) superimposed upon a binary average image of the TCR microclusters (CD3ζ) are shown in light colors for those inside the microclusters and in dark colors for those outside the microclusters. Bar, 1 μm. (C–F) The PDF of CD3ε (C, E) and CD45 (D, F) inside (C, D) and outside (E, F) the microclusters during Δt = 1 frame (33.33 ms) is fitted with a two-state diffusion model PDF_2 (Equation S1) (solid line). The dotted lines represent the component PDFs of the slower states (red) and faster states (blue).

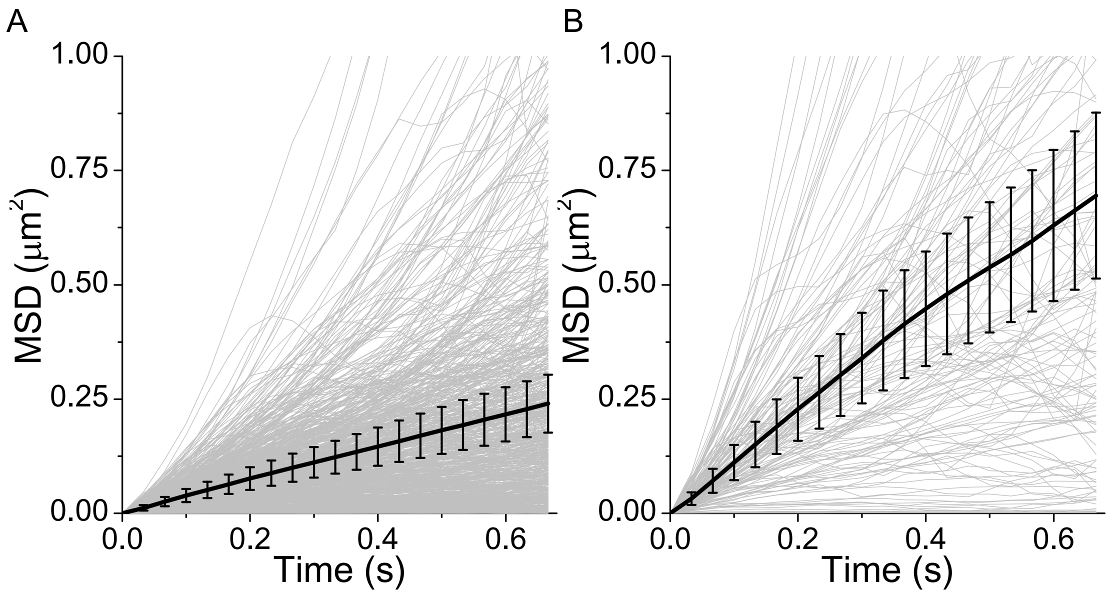

Figure S2. (A, B) Standard MSD analysis of single-molecule trajectories of CD3ε (A) and CD45 (B) shown as a function of time. Black line: ensemble-averaged MSD curves with error bars representing standard errors of means; gray line: MSD curves of individual trajectories.

Supplementary Tables

Table S1. Diffusion coefficients D obtained by standard single-molecule tracking analysis[a]

MSD analysis (Equations 1 and 2)

	D (μm^2/s)	N_{traj}
CD3ε	0.0911 (± 0.0006)	775
CD45	0.270 (± 0.004)	162

PDF analysis using the two-state diffusion model (Equation S1)

		Slow		Fast		N_{step}
		D_{slow} (μm^2/s)	ξ[a] (%)	D_{fast} (μm^2/s)	$1-\xi$[a] (%)	
CD3ε	Outside	0.0062 (± 0.0009)	21 ± 2	0.090 (± 0.007)	79 ± 2	37,536
	Inside	0.0041 (± 0.0006)	34 ± 3	0.057 (± 0.004)	66 ± 3	14,738
CD45	Outside	0.0238 (± 0.0053)	15 ± 2	0.258 (± 0.016)	85 ± 2	9,801
	Inside	0.0091 (± 0.0015)	23 ± 2	0.178 (± 0.016)	77 ± 2	2,222

Data, fitted value ± 95% confidence interval. N_{traj} indicates the number of trajectories, and N_{step} indicates the number of steps from two cells.

[a] The relative occurrences of the slower and faster diffusion states are ξ and $1 - \xi$, respectively.

Table S2. Lifetimes of trajectory durations and residence times obtained by moving subtrajectory analysis (Fig. 4)

Lifetime of trajectory durations (Equation 10)

	τ_{traj_short} (s)	ξ^{a} (%)	τ_{traj_long} (s)	$1-\xi^{a}$ (%)	N_{traj}
CD3ε	0.25 (± 0.01)	45 (± 2)	0.91 (± 0.02)	55 (± 2)	1,521
CD45	0.46 (± 0.01)	86 (± 2)	3.19 (± 0.57)	14 (± 2)	302

Lifetime of residence times (Equation 11)

	CD3ε		CD45	
	τ (s)	N_{res_*}[b]	τ (s)	N_{res_*}[b]
τ_{res_out}	1.92 (± 0.05)	1,131	1.18 (± 0.04)	287
τ_{res_bou}	0.54 (± 0.04)	962[c]	0.68 (± 0.06)	245[c]
$\tau_{res\_bou\to out}$	0.50 (± 0.03)	793	0.64 (± 0.05)	245
$\tau_{res\_bou\to in}$	0.43 (± 0.02)	654	0.38 (± 0.02)	136
τ_{res_in}	1.52 (± 0.11)	368	1.15 (± 0.16)	31

Ratio of residence times

	CD3ε	CD45
$\tau_{res\_out} / \tau_{res\_in}$	1.27 (± 0.10)	1.03 (± 0.14)
$\tau_{res\_bou\to in} / \tau_{res\_bou\to out}$	0.87 (± 0.07)	0.60 (± 0.06)
$N_{rest\_bou\to in} / N_{res\_bou\to out}$	0.83	0.56

Data, fitted value with ± 95% confidence interval. N_{traj} and $N_{subtraj}$ indicate the number of trajectories and subtrajectories, respectively, from two cells.

[a] The relative occurrences of the shorter and longer lifetimes are ξ and $1 - \xi$, respectively.

[b] N_{res_in}, N_{res_bou}, and N_{res_out} are the number of data elements.

[c] The reason that $N_{subtraj}$ of τ_{res_bou} is not the sum of $N_{subtraj}$ of $\tau_{res\_bou\to out}$ and that of $\tau_{res\_bou\to in}$ is that both contain common subtrajectories that are terminated by the end of the trajectories rather than by exiting to the inside or outside.

Table S3. Diffusion coefficients of the slower and faster mobility states obtained by moving subtrajectory analysis using Equations 2, 6, and 7 (Fig. 5A and B)

			Slower		Faster		$N_{subtraj}$
			D_{slow} (μm^2/s)	ξ^a (%)	D_{fast} (μm^2/s)	$1-\xi^a$ (%)	
CD3ε	Outside	Overall[b]	0.0024 (+0.0006 / −0.0004)	12 (± 1)	0.095 (± 0.002)	88 (± 1)	16,275
		Directional	0.0013 (± 0.0001)	24 (± 1)	0.070 (± 0.001)	76 (± 1)	7,201
		Free	0.0069 (± 0.0002)	55 (± 2)	0.078 (± 0.003)	45 (± 2)	1,790
		Confined		-	0.115 (± 0.002)	-	7,284
	Boundary	Overall[b]	0.009 (+0.043 / −0.008)	6 (± 2)	0.094 (± 0.002)	94 (± 2)	7,908
		Directional	0.007 (+0.010 / −0.004)	9 (± 2)	0.072 (± 0.002)	91 (± 2)	3,685
		Free	0.008 (± 0.001)	52 (± 2)	0.083 (± 0.004)	48 (± 2)	819
		Confined	-	-	0.118 (± 0.003)	-	3,404
	Inside	Overall[b]	0.0025 (+0.0004 / −0.0003)	50 (± 3)	0.066 (+0.007 / −0.006)	50 (± 3)	5,229
		Directional	0.0014 (± 0.0001)	80 (± 4)	0.044 (+0.009 / −0.007)	20 (± 4)	2,795
		Free	0.0067 (± 0.0001)	91 (± 2)	0.034 (+0.013 / −0.010)	9 (± 2)	976
		Confined	-	-	0.067 (± 0.003)	-	1,458
CD45	Outside	Overall[b]	0.0030 (+0.0004 / −0.0003)	16 (± 3)	0.225 (± 0.007)	84 (± 3)	3,879
		Directional	0.0026 (± 0.0002)	27 (± 4)	0.171 (± 0.006)	73 (± 4)	1,643
		Free	0.0041 (± 0.0001)	60 (± 3)	0.201 (+0.015 / −0.014)	40 (± 3)	342
		Confined	-	-	0.287 (± 0.009)	-	1,894
	Boundary	Overall[b]	0.0059 (+0.0043 / −0.0025)	5 (± 3)	0.249 (± 0.008)	95 (± 3)	2,560
		Directional	0.0028 (+0.0009 / −0.0007)	8 (± 5)	0.185 (+0.007 / −0.006)	92 (± 5)	1,084
		Free	0.0067 (+0.0008 / −0.0007)	38 (± 6)	0.225 (+0.022 / −0.020)	62 (± 6)	249
		Confined	-	-	0.349 (+0.017 / −0.016)	-	1,227
	Inside	Overall[b]	0.0015 (± 0.0002)	54 (± 5)	0.14 (± 0.02)	46 (± 5)	469
		Directional	0.0013 (± 0.0001)	86 (± 7)	0.08 (+0.04 / −0.02)	14 (± 7)	246
		Free	0.0047 (± 0.0002)	83 (± 6)	0.11 (± 0.03)	17 (± 6)	57
		Confined	-	-	0.17 (± 0.01)	-	166

Data, fitted value with ± 95% confidence interval. $N_{subtraj}$ indicates the number of subtrajectories from two cells.

[a] The relative occurrences of the slower and faster diffusion states are ξ and $1 - \xi$, respectively.

[b] Overall distributions, which were the total of the three diffusion types, were used for fitting.

Table S4. Velocities of the directional diffusion v_{direc} and confinement radii r_{conf} obtained by moving subtrajectory analysis (Fig. 5C–F)

The velocity of directional movement v_{direc} (Equation 6)

		Slower		Faster		$N_{subtraj}$
		v_{direc_slow} (μm)	ξ[a] (%)	v_{direc_fast} (μm)	$1-\xi$[a] (%)	
CD3ε	Outside	0.0264 (+0.0006 / −0.0005)	34 (± 1)	1.10 (± 0.01)	66 (± 1)	7,284
	Boundary	0.0295 (+0.0065 / −0.0053)	11 (± 4)	1.18 (± 0.03)	89 (± 4)	3,685
	Inside	0.0263 (+0.0007 / −0.0006)	84 (± 2)	0.55 (+0.11 / −0.09)	16 (± 2)	1,458
CD45	Outside	0.036 (+0.007 / −0.006)	16 (± 4)	1.80 (± 0.07)	84 (± 4)	1,894
	Boundary	0.038 (+0.020 / −0.013)	4 (± 3)	1.85 (± 0.04)	96 (± 3)	1,227
	Inside	0.031 (± 0.002)	78 (± 7)	0.90 (+0.15 / −0.13)	22 (± 7)	166

The confinement radius r_{conf} (Equations 7 and 8)

	Outside		Boundary		Inside	
	r_{conf} (μm)	$N_{subtraj}$	r_{conf} (μm)	$N_{subtraj}$	r_{conf} (μm)	$N_{subtraj}$
CD3ε	0.187 (± 0.002)	7,201	0.198 (± 0.002)	3,404	0.114 (± 0.005)	2,795
CD45	0.299 (± 0.004)	1,646	0.346 (± 0.005)	1,085	0.199 (+0.16 / −0.15)	246

Data, fitted value with ± 95% confidence interval. $N_{subtraj}$ indicates the number of subtrajectories from two cells.

[a] The relative occurrences of the slower and faster diffusion states are ξ and $1-\xi$, respectively.

Table S5. Association and dissociation rates of the transitions between the faster diffusion (dissociated) states and the slower diffusion (associated) states (Fig. 6)

Association rates using Equations 12 and 13

	CD3ε		CD45	
	k_{on} (s^{-1})	$N_{subtraj}$	k_{on} (s^{-1})	$N_{subtraj}$
Overall[b]	0.71 (± 0.02)	1,763	0.179 (± 0.007)	317[c]
Outside	0.58 (± 0.02)	1,210	0.20 (± 0.03)	294
Boundary	0.49 (± 0.14)	989	0.95 (± 0.14)	268
Inside	3.1 (± 0.2)	358	2.7 (± 0.5)	27

Dissociation rates using Equations 14 and 15

		Slower		Faster		$N_{subtraj}$
		k_{off_slow} (s^{-1})	ν[a] (%)	k_{off_fast} (s^{-1})	$1-\nu$[a] (%)	
CD3ε	Overall[b]	0.00 (± 0.04)[d,e]	41 (± 1)	10.8 (± 0.6)	59 (± 1)	718[c]
	Outside	0.0 (± 0.1)[d,e]	43 (± 4)	12 (± 2)	57 (± 4)	370
	Boundary	1.6 (± 1.0)	50 (± 20)	18 (± 8)	50 (± 20)	180
	Inside	0.0 (± 0.1)[d,e]	81 (± 3)	45 (± 34)	19 (± 3)	246
CD45	Overall[b]	0.00 (± 0.09)[d,f]	36 (± 4)	61 (± 65)	36 (± 4)	37[c]
	Outside	0.0 (± 0.7)[d,f]	60 (± 10)	88 (± 312)	40 (± 10)	18
	Boundary	0.0 (± 0.4)[d,f]	70 (± 10)	24 (± 15)	30 (± 10)	17
	Inside	0 (± 1×10^{2})[d,f]	100 (± 100)[g]	0 (± 2×10^{8})[h]	0 (± 100)[g]	9

Data, fitted value with ± 95% confidence interval. $N_{subtraj}$ indicates the number of subtrajectories from two cells.

[a] The relative occurrences of the faster and slower transitions are ν and $1-\nu$, respectively.

[b] Overall data were obtained and analyzed without location classification.

[c] The reason why overall $N_{subtraj}$ is not the sum of those on the outside, boundary, and inside is that they contain common subtrajectories that are terminated at the end of the trajectories or by exit to another region, not by association or dissociation.

[d] The rate k_{off_slow} could not be determined by the fitting due to the large relative value of the standard deviation of the fit parameter. This means that k_{off_slow} is much less than the inverse of the trajectory lifetime, which is involved in Equations 14 and 15.

[e] CD3ε: $k_{off\_slow} \ll 1.09\ s^{-1}$, $1/k_{off\_slow} \gg 0.91$ s.

[f] CD45: $k_{off\_slow} \ll 0.31\ s^{-1}$, $1/k_{off\_slow} \gg 3.2$ s.

[g] The large error for CD45 was caused by the small number of subtrajectories; as judged by the decay curve, the slower fraction is in a great majority.

[h] The rate could not be determined due to the small fraction of the small number $N_{subtraj}$.
